# Sexual dimorphic parameters of femur: a clinical guide in orthopedics and forensic studies

**DOI:** 10.25122/jml-2021-0022

**Published:** 2021

**Authors:** Shajiya Sarwar Moosa, Mohammad Habeebur Raheman Shaikh, Moizuddin Khwaja, Siraj Ahmed Hasham Shaikh, Faiza Banu Siddiqui, Syed Rehan Hafiz Daimi, Sanket Dadarao Hiware, Essam Elbadawy Ismail, Yousuf Begum

**Affiliations:** 1. Department of Anatomy, College of Medicine, Imam Abdulrahman Bin Faisal University, Dammam, Saudi Arabia; 2. Department of Physiology, College of Medicine, Imam Abdulrahman Bin Faisal University, Dammam, Saudi Arabia

**Keywords:** femur, sexual dimorphism, maximum length of femur, trochanteric oblique length, vertical diameter of the head, L – Maximum length of femur, TOL – Trochanteric oblique length, VDH – Vertical diameter of the head

## Abstract

Sexual dimorphic studies of various parameters of the femur play an important role in forensic studies. Various femur morphometric parameters help estimate an individual’s age, sex, and stature from unknown skeletal remains. This research was done to analyze maximum length, trochanteric oblique length, and diameter of the femur head for sexual dimorphism. The study was done on 200 (128 male & 72 female) Indian adult human femora, which were fully ossified, dry, and free from deformity. The maximum length of the femur (L), trochanteric oblique length (TOL), and vertical diameter of the head (VDH) were measured using an osteometric board and digital Vernier calipers. The mean length of the femur was 436.88 mm in males and 402.38 mm in females, respectively. The mean trochanteric oblique length of the femur was 423.78 mm in males and 387.18 mm in females, respectively. The mean vertical diameter of the femur head was 43 mm in males and 38.19 mm in females, respectively. Depending upon the results of this study, it was concluded that the mean values of maximum length, trochanteric oblique length, and vertical diameter of the femur head are significantly higher in males than females. These parameters are useful and reliable for sexual dimorphism in anthropometric and forensic studies, especially in identifying skeletal remains. These differences can also be considered in selecting or designing the exact ranges of the gender-specific prosthesis for Orthopedic surgeries.

## Introduction

The hip joint is very stable and it is the largest joint of the body. This specific feature is governed by the typical anatomical shape of articulating surfaces and ligaments. It is a multiaxial, ball, and socket joint. Its maximum stability is due to the deep insertion of the head of the femur into the acetabulum [1]. The femur is one of the largest bones of the body subjected to maximum weight-bearing; its typical geometric shape gives it strength and stability. Morphometric parameters, including hip axis length, femoral head width, have been related to the mechanical strength of the proximal femur [2].

The morphology of the proximal femur, especially the relationships between the head, neck, and the proximal shaft, has been investigated numerous times. There are many pathologies like avascular necrosis, osteoporotic fractures, osteoarthritis etc, and a greater understanding of the anatomy of this area might refine treatment options for these conditions [3]. Consequently, researchers started studying the measurements of the proximal femur. Many forensic studies have proved the importance of various morphometric parameters of the femur, which help to estimate biological profiles including age, sex, ancestry, and stature of an individual to identify unknown skeletal remains.

The dimensions of the head and length of the femur were studied extensively by many researchers, and conclusions were drawn. It was found to differ in different population groups and at different ages; the findings on sex were also slightly different [4]. 

Information on the variations in dimensions of the femur for different sexes will help the Anatomists and Forensic experts in sexing the femora. Moreover, it will help in devising proper sized prosthesis. It will also help Orthopedic Surgeons in femoral head replacement surgeries. Furthermore, awareness regarding gender differences will lead to distinct implant designs for male and female patients [5]. Considering the factors above, this study was undertaken to determine the normal range of metrical values for length and measurement of the femur head in males and females on adult human cadaveric bones. Knowing the importance of sexual dimorphic studies in calibrating exact sized prosthesis in Orthopedics and identification of skeletal remains for Forensic experts, the present study aims:

1. To study and analyze maximum length, trochanteric length, and diameters of the head of the femur for sexual dimorphism;
2. To compare femoral sexual dimorphic findings of the present study with that of other studies.

## Material and Methods

200 skeletonized samples (128 male and 72 female) adult human femur of known sex, dry, free from deformity, and fully ossified were obtained from the bone bank of Department of Anatomy, Government Medical College, Aurangabad, Maharashtra, India. The samples were taken from the year 1995–2018. The sex and year of samples collection were well documented in the bone bank. The samples were obtained by the burial method. The anthropometric tools used in the study were: osteometric board, sliding digital calipers, and scale. The inclusion criteria were dry, free from deformity, and fully ossified adult human femora. The exclusion criteria were damaged, burnt, abnormal bones and bones of children 

**Figure 1.**
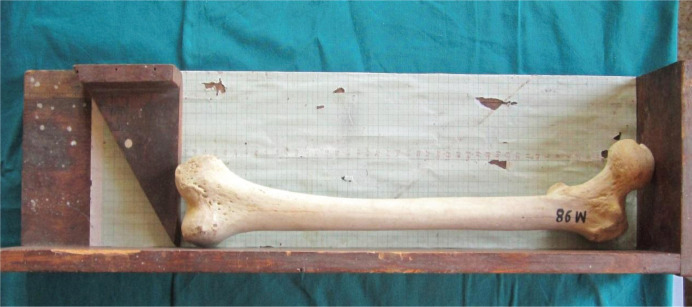
Measurement of maximum length of the femur.

**Figure 2.**
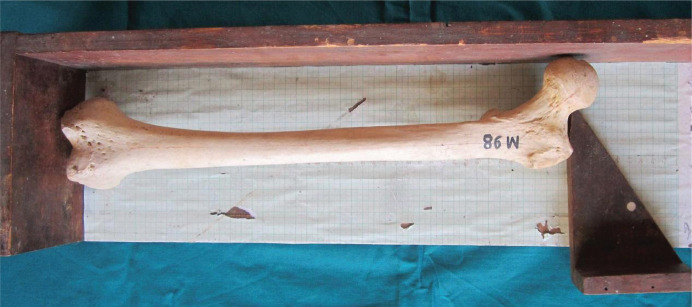
Measurement of trochanteric oblique length.

**Figure 3.**
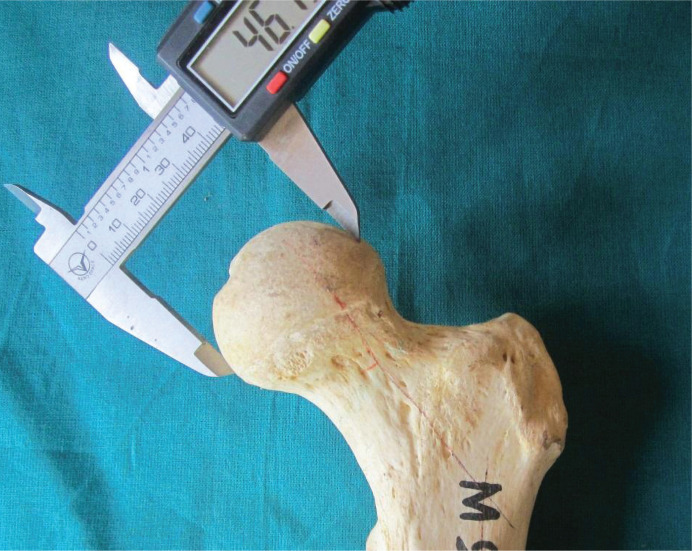
Measurement of vertical diameter of the head.

The following measurements were taken as described by Singh I.P. [6] and M.Sreenivas [4]:

1. Maximum length of the femur (L): measures the distance from the highest point of the head to the infra-condylar plane (Figure 1);
2. Trochanteric oblique length (TOL): measured as the vertical distance from the top of the greater trochanter to the infra-condylar plane (Figure 2). The above two measurements were taken using an osteometric board;
3. Vertical diameter of the head (VDH): measured as the straight distance between the highest (proximal) point of the head to the deepest (distal) point on the inferior aspect of the head (Figure 3).

The above measurements were taken using sliding digital Vernier calipers. Each parameter was tabulated and statistically analyzed. Mean, standard deviation, and ranges were obtained for male and female femora, and an independent t-test was applied for statistical analysis. For statistical analysis, GraphPad Prism 5.01 software was used. Comparative graphs of male and female values were drawn, which show the zone of difference and overlap between male and female values.

## Results

**Figure 4.**
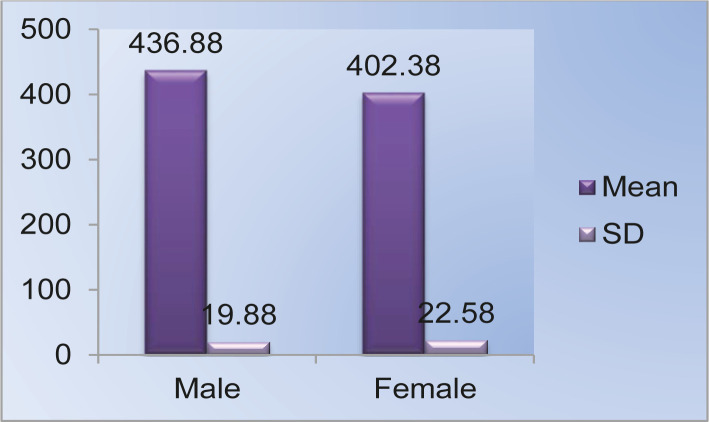
Mean and standard deviation maximum length (mm).

The mean value for the maximum length of the femur was 436.88 mm in males and 402.38 mm in females, with a range of 392–490 mm and 360–469 mm, respectively. The standard deviation in males was 19.880 and 22.581 in females. In comparison, the mean values in males and females were highly significant (p-value <0.001) (Figure 4, Table 1). 

**Table 1.** Mean values and statistical analysis of length, trochanteric oblique length, diameter of the femur head in males and females.

**Parameters**	**Maximum length (mm)**		**Trochanteric length (mm)**		**Vertical diameter of the head (mm)**	
	Male	Female	Male	Female	Male	Female
**Range**	392–490	360–469	386–471	303–452	30-54-48.73	33.33–43.34
**Mean**	436.88	402.38	423.78	387.18	43	38.19
**SD**	19.880	22.581	19.024	40.152	2.4185	2.3040
**Mean±3SD**	377.24–496.52	334.64–470.123	366.71–480.85	266.72–507.64	35.74–50.26	31.28–45.102
**t value**	11.21		8.72		13.71	
**p value**	<0.001(HS)		<0.001(HS)		<0.001(HS)	

**Figure 5.**
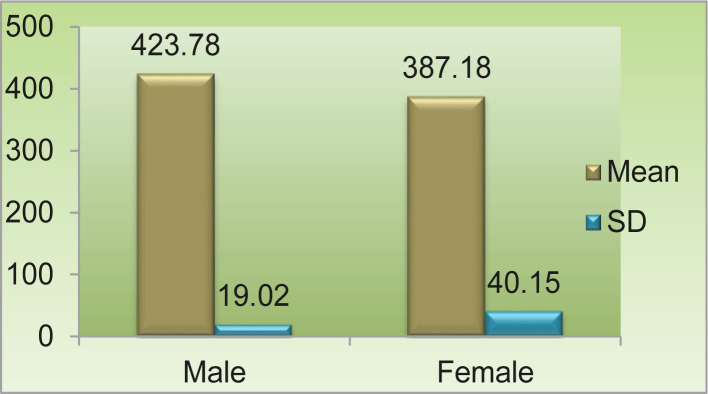
Mean and standard deviation trochanteric oblique length (mm).

The mean value for the trochanteric oblique length of the femur was 423.78 mm in males and 387.18 mm in females with a range of 386–471 mm and 303–452 mm, respectively. The standard deviation in males was 19.024, and 40.152 in females. In comparison, the mean values in males and females were highly significant (p-value <0.001) (Figure 5, Table 1). 

**Figure 6.**
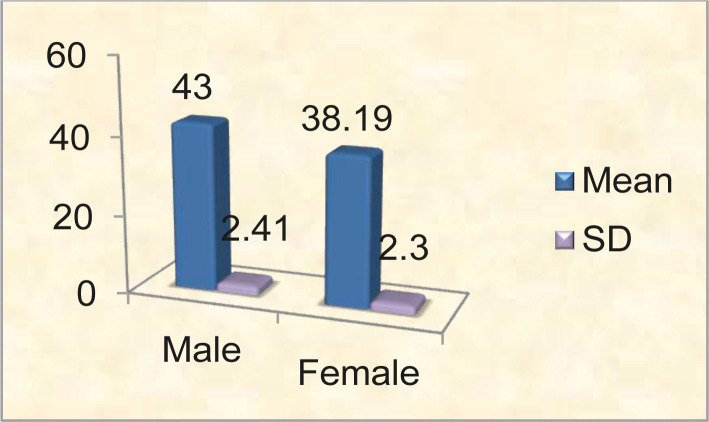
Mean and standard deviation vertical diameter of the head (mm).

The mean value of the vertical diameter of the femur head was 43 mm in males and 38.19 mm in females, with a range of 30-54-48.73 mm and 33.33–43.34 mm, respectively. The standard deviation in males was 2.4185 and 2.3040 in females. In comparison, the mean values in males and females were highly significant (p-value <0.001) (Figure 6, Table 1). 

## Discussion

In the preceding discussion, these results are compared with those of previous researchers. The mean value for the total length of the femur was 436.88 mm in males and 402.38 mm in females, with a range of 392–490 mm and 360–469 mm, respectively. The standard deviation in males was 19.880 and 22.581 in females. In comparison, the mean values in males and females were highly significant (p-value <0.001). Studies conducted by other researchers were in concordance with the findings of our study. For example, R. Purkait and Chandra in their study performed on 200 male and 80 female femora, found the mean value of the total length of the femur to be 451.47 mm in males and 403.69 mm in females. Comparing the mean values in males and females, they found that total length was higher in males than in females [7].

Similarly, the study carried out by Gargi Soni *et al.* on 40 male and 40 female femora showed the mean value of the total length of the femur to be 439.57 mm in males and 410.60 mm in females with a high significance statistically [8]. Kalpana R *et al.* made a similar study on 100 males and 100 females and found the mean value of the total length of femora to be 441.36 mm and 394.60 mm, having significant results for male and female respectively [9]. However, other studies showed the mean value of the total length of the femur in both males and females a little different as compared to the present study [10–14]. The mean value for the trochanteric oblique length of the femur was 423.78 mm in males and 387.18 mm in females with a range of 386–471 mm and 303–452 mm, respectively. The standard deviation in males was 19.024 and in females, 40.152.

In comparison, the mean values among males and females were highly significant (p-value <0.001). Leelavanthy *et al.* obtained the mean values for trochanteric oblique length in the right and left femora of males 419.3 mm and 421.5 mm and 389.8 mm and 385.6 mm in the right and left femora of females, respectively. Comparing the mean values in males and females, they found that trochanteric oblique length was higher in males than in females on both sides. However, it was found that the values were not statistically significant for the right side but highly significant for the left side [15]. In a study conducted by Shital M. *et al.* on 187 male and 179 female samples, the mean value for trochanteric oblique length was 440.3 mm and 396.4 mm, respectively [12]. However, the studies conducted by Shital M. *et al.* [12], P.S. Igbigbi and B.C. Msamati [16] and Pearson [17] showed the mean value of trochanteric oblique length on the higher side in both males and females compared to the present study. The mean value for the vertical diameter of the femur head was 43 mm in males and 38.19 mm in females, with a range of 30-54-48.73 mm and 33.33–43.34 mm, respectively. The standard deviation in males was 2.4185 and 2.3040 in females. In comparison, the mean values in males and females were highly significant (p-value <0.001). 

The results of the head of femur of the present study were found to be correlating well with the observations made by other researchers [9, 12, 18]. Shital M. *et al.* in their study of 187 male and 179 female femora, found the mean value of the vertical diameter of the head of the femur to be 43.61 mm in males and 38.7 mm in females. Comparing the mean values in males and females, they found the vertical diameter higher in males than females and was statistically highly significant [12]. Similarly, Kalpana R. *et al.* in their study on 100 male and 100 female femora, found the mean value of the vertical diameter of the head of the femur to be 44.37 mm in males and 38.44 mm in females. Comparing the mean values in males and females, they found that the vertical diameter of the head was higher in males than in females and was statistically highly significant [9]. In 2019, a similar study was conducted by K.V. Pavan Kumari and found values higher in males [19]. The studies done by other authors showed the mean values of the parameter under discussion to be a little different as compared to the present study [10, 16, 20]. The present study is in concordance with most of the studies. However, sample size and use of one measurement method remain the main limitations.

## Conclusion

Focusing on the results of this study, it was concluded that the mean values of maximum length, trochanteric oblique length, and vertical diameter of the head of the femur are significantly higher in males than females. These parameters are useful and reliable for sexual dimorphism in anthropometric and Forensic studies, especially in identifying skeletal remains. These differences can also be considered in selecting or designing the exact ranges of gender-specific prostheses for Orthopedic surgeries.

## Acknowledgements

### Conflict of interest

The authors declare that there is no conflict of interest.

### Ethics approval

The study was reviewed and approved by the Institutional Ethics Committee, ID number SDA/UE/HF/12.

### Personal thanks

We are highly indebted and would like to place on record our gratitude to the Government Medical College & Hospital, Aurangabad, and Department of Anatomy for providing an opportunity and facilities for the study.

### Authorship

SSM devised the concept and design of this study, conducted literature search, prepared manuscript, and analyzed data. MHS helped in conceptualizing, editing, and reviewing this manuscript. SDH helped in analysis and critical review. The rest of the authors made significant contributions in revising and reviewing the article as well as giving final approval of the draft.
